# Assessing public health threats: An overview of coordinated threat assessment at the Public Health Agency of Canada

**DOI:** 10.14745/ccdr.v52i05a02

**Published:** 2026-05-31

**Authors:** Gabrielle Brankston, Ekim Bagree, Rukshanda Ahmad, Camille Dulong, Catherine Elliott, Eleni Galanis, Jacqueline Middleton, Amrit Sandhu, Lindsay Whitmore

**Affiliations:** 1 Applied Public Health Sciences Directorate, Public Health Agency of Canada, Ottawa, ON; 2 School of Population and Public Health, University of British Columbia, Vancouver, BC

**Keywords:** public health, threat assessment, tool, method, framework, public health intelligence

## Abstract

**Background:**

The early detection, assessment, and communication of potential public health threats are key activities in mitigating public health impacts by ensuring the timely deployment of an appropriate response. The Public Health Agency of Canada (PHAC) uses a standardized methodology to consolidate scientific and technical subject matter expertise on a weekly basis to systematically characterize and assess emerging public health signals and communicate assessments to decision-makers.

**Methods:**

Public Health Agency of Canada’s coordinated threat assessment process, and the standardized methodology used to characterize and assess threats, are described.

**Results:**

The coordinated threat assessment methodology uses a standardized method that includes engagement with subject matter experts across PHAC to assess, document, and communicate the characterization of each threat. Three key attributes are used to assess each threat based on the concern for human health, the potential to affect Canada, and the concern that Canada has insufficient control measures to manage a specific threat. Qualitative criteria for each threat attribute are used to assess a threat based on ‘high’, ‘moderate’, or ‘low’ levels. An overall assessment is based on the three threat attributes and is accompanied by a narrative rationale to support the assigned threat level.

**Conclusion:**

The standardized threat assessment methodology provides a coordinated and systematic process to characterize and assess public health threats in a consistent and timely manner. The framework reflects important elements to consider in terms of public health significance, allows for flexibility in the indicators used to characterize a public health threat, and is applicable to any threat of public health concern.

## Introduction

Public health intelligence is a core public health function responsible for detecting, assessing, interpreting, and communicating information for informed decision-making to protect the health of the population ((1)). These activities are key in mitigating public health impacts by ensuring the timely deployment of an appropriate response, and are in place in subnational, national, and international public health agencies ((2–4)). Some agencies follow a centralized approach, wherein a core team is responsible for all steps, including signal detection, assessment, and communication ((2,4)). Others follow a disseminated approach, with responsibility for these activities distributed among a variety of teams across the agency ((5)).

As part of the spectrum of public health intelligence activities, threat assessment is a high-level, early characterization of the public health significance associated with a specific signal or event ((4)) using data and intelligence from multiple sources, as well as expert opinion ((6)). Indicators of public health significance are measured in different ways by different public health agencies, however, factors such as population health impact, potential for exposure or transmission, and the availability of public health control measures are often considered ((2–4)). The threat assessment process can be challenging due to limited or emerging data, however, a standardized methodology ensures all public health threats are characterized using a consistent approach to inform a public health response that is proportionate to the threat level ((6)).

At the Public Health Agency of Canada (PHAC), threat assessment is standardized through the coordinated threat assessment (CTA) process, which was established in response to recommendations identified by the Auditor General of Canada in 2021 to strengthen its risk assessment process and coordination to guide public health response ((7)). Situated within a spectrum of risk assessment activities, CTA is a PHAC-wide approach that consolidates data, information, and subject matter expertise on a weekly basis to systematically assess emerging public health signals that may constitute a threat to Canada and potentially require further public health action. Through the CTA process, a weekly threat report is produced and shared with public health partners to document and communicate an early, high-level assessment of emerging public health threats. This article provides an overview of PHAC’s CTA process and a description of the methodology used to assess threats. **Table 1** contains a list of terms associated with threat assessment and their definitions.

**Table 1:** Public Health Agency of Canada definitions of technical terms associated with public health threat assessment

Technical term	Definition
Hazard	Anything with the potential to cause harm ((8)).
Signal	Data and/or information representing a potential acute risk to human health. Signals may consist of reports of cases or deaths (individuals or aggregated) or potential exposure of human beings to hazards ((9)).
Event	A signal that has been assessed and verified ((9)).
Threat	An event that has been further assessed to have the potential (directly or indirectly) to cause harm to Canadians or individuals living in Canada and may require further action ((10)).
Risk	A function of the likelihood that a public health event or threat may occur and the magnitude of the impact if it were to occur (11)).

## Methods

### Coordinated threat assessment methods

#### Overall process

**Figure 1:**
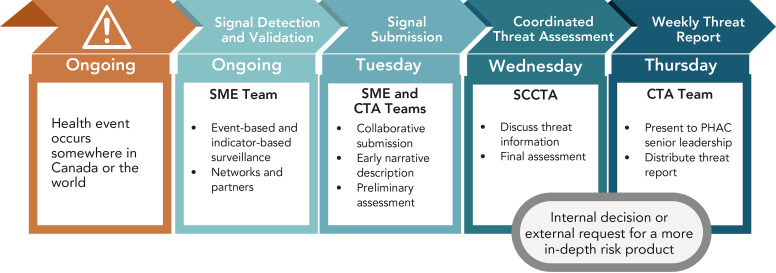
Overview of Public Health Agency of Canada’s weekly coordinated threat assessment cycle Abbreviations: CTA, coordinated threat assessment; PHAC, Public Health Agency of Canada; SCCTA, Scientific Committee for Coordinated Threat Assessment; SME, subject matter experts

The threat assessment process begins when PHAC detects a signal indicating a potential hazard to human health occurring either in Canada or internationally (**Figure 1**). Signals of potential public health threats may be related to a new hazard or a change in the epidemiology of an existing hazard. This includes, but is not limited to, an increase in morbidity or mortality in animals or humans, a change in the geographical or temporal distribution of a given hazard, an unexpected cluster of cases or deaths, the emergence or re-emergence of a pathogen, and hazards that have the potential to cause substantial morbidity or mortality in humans.

**Signal detection and validation:** Signals are detected through a variety of sources, including notification from subnational, national, and international partners, as well as event-based surveillance or indicator-based surveillance systems ((6,12)). Subject matter experts (SMEs) within PHAC validate signals to determine whether they have the potential to constitute a threat to Canada. The threat assessment process is initiated for signals that meet the specific submission criteria described in the threat assessment framework, described below.

**Assessment:** A preliminary threat assessment is provided by the submitting SME in collaboration with the CTA team using the standardized methodology. The final threat assessment is determined at a weekly meeting of the Scientific Committee for Coordinated Threat Assessment (SCCTA), which consists of scientific and technical public health experts with a wide breadth of expertise across PHAC. The consolidation of expert opinion using a standardized assessment tool results in a transparent and systematic measure of the public health threat level. Additional risk assessments are considered for complex threats or those identified by SMEs to require a more in-depth assessment of risk ((13)). Threats are reassessed on a weekly basis as a situation evolves, and more information becomes available.

**Communication and documentation:** Timely and clear communication of the threat level to PHAC senior leadership leads to a well-informed and common understanding of the threat to support decision-making for further action. The weekly threat report documents the signal, the assessment and rationale, as well as the public health actions undertaken by PHAC to address the threat.

A central team coordinates the process and is composed of epidemiologists and a medical director. Lead and supporting PHAC programs associated with each threat select a representative to contribute to the written submission and oral presentation at SCCTA. Subject matter experts in a variety of areas, including risk assessment, convene at SCCTA meetings to discuss and finalize the threat assessment for each submission.

## Results

### Threat assessment framework

**Figure 2:**
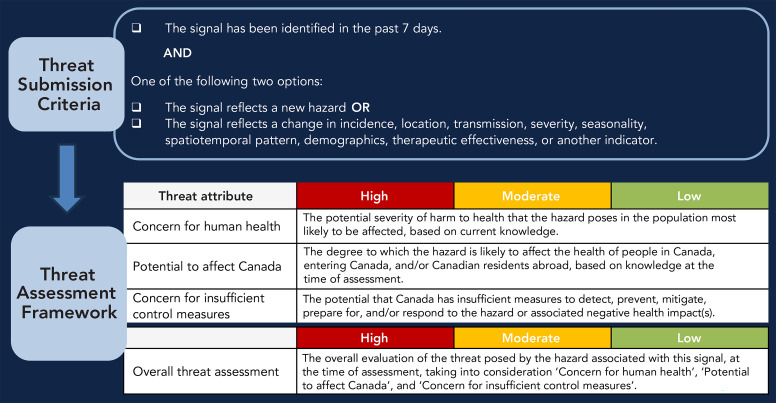
Threat submission and assessment framework

Threat assessment at PHAC utilizes a standard framework that involves assessment of three threat attributes that characterize the public health significance of the event, and an overall threat level associated with the event (**Figure 2**) ((14)). The framework can be used to characterize threats affecting the general population or a specific subgroup, which may include populations at increased risk of severe harm due to biology (e.g., immune compromise, age), socioeconomic factors (e.g., access to care), or geography. Uncertainty in the data used to characterize a threat may elevate the assessment level of any of the attributes or the overall assessment.

#### Threat attributes

Each of the threat attributes is individually assessed and assigned a level of ‘high’, ‘moderate’, or ‘low’ based on the definitions in **Table 2**. These definitions allow for flexibility in the indicators used to characterize a threat and can be applied to any public health threat.

**Table 2:** Definitions associated with each threat attribute

Threat attribute	High	Moderate	Low
Concern for human health	This hazard poses or is anticipated to pose severe harm to the health of the population most likely to be affected (e.g., a hazard that is typically life-threatening and/or involves permanent or long-term harmful health consequences).	This hazard poses or is anticipated to pose moderate harm to the health of the population most likely to be affected (e.g., a hazard that is typically associated with serious but not life-threatening health effects or typically involves non-permanent or short-term harmful health consequences).	This hazard poses or is anticipated to pose minor harm to the health of the population most likely to be affected (e.g., a hazard that is typically self-limiting or associated with minimal harmful health consequences).
Potential to affect Canada	The hazard is currently affecting or is likely to affect large numbers of people in the general population or in specific subgroups in Canada, entering Canada, and/or Canadian residents abroad.	The hazard is currently affecting or is likely to affect a moderate number of people in the general population or in specific subgroups in Canada, entering Canada, and/or Canadian residents abroad.	The hazard is currently affecting or is likely to affect a minimal number of people in the general population or in specific subgroups in Canada, entering Canada, and/or Canadian residents abroad. OR The hazard is unlikely to affect people in Canada, entering Canada, and/or Canadian residents abroad.
Concern for insufficient control measures	There are currently no known mechanisms to detect, prevent, mitigate, prepare for, and/or respond to the impact(s) of this hazard on Canada^a^. OR There may be limited mechanisms to detect, prevent, mitigate, prepare for, and/or respond to the impact(s) of this hazard on Canada^a^, but they are considered experimental, of unknown effectiveness, or unavailable. OR Necessary measures will require significant resources to implement.	There are mechanisms to detect, prevent, mitigate, prepare for, and/or respond to the impact(s) of this hazard on Canada^a^, but there may be challenges with effectiveness, availability, or deployment. OR Necessary measures may require increased resources to implement.	There are effective, available, and easily deployed mechanisms to detect, prevent, mitigate, prepare for, and/or respond to the impact(s) of this hazard on Canada^a^. OR Routine responses are adequate (i.e., there is no need to implement additional control measures).

^a^ Canada refers to people living in Canada, entering Canada, and/or Canadian residents abroad

**Concern for human health:** Reflects the severity of harm posed to human health (Table 2). Indicators to consider for this attribute include morbidity, mortality, case fatality ratio, and burden of harm as it relates to physical, mental, or social well-being. When assessing concern for human health, the health consequences that are typically associated with the population most likely to be affected are considered. The level assigned for this attribute may vary depending on the context of the situation being assessed. For example, an outbreak of an enteric pathogen mainly involving populations at high risk of severe illness (e.g., children under the age of five years) may be assigned a higher level than the same pathogen affecting a group of young and middle-aged adults.

**Potential to affect Canada:** Describes the number of individuals or proportion of a population that will potentially be affected by a hazard (Table 2). Examples of indicators to consider include the transmissibility of a pathogen, the distribution of disease vectors in Canada, and the geographical range of a hazard, such as wildfire smoke. The number of people potentially or currently affected that would be considered high, moderate, or low will vary depending on the context of the event. For example, an assessment of ‘high’ may refer to a large proportion of a particular subgroup within Canada or a large number of people in the general population.

**Concern for insufficient control measures:** Relates to Canada’s capacity to engage in public health activities to detect or respond to a hazard (Table 2). Activities related to detection, prevention, mitigation, preparation, or response may be applicable at different stages of an event. For example, detection refers to whether Canada has sufficient surveillance and/or laboratory capacity to detect a hazard should it enter Canada and should be considered in the assessment of threats that have not yet entered Canada. In contrast, prevention and response refers to whether Canada has mechanisms, such as vaccines, therapeutics, safety protocols, and guidelines to prevent the spread of the hazard. In addition, some control measures are dependent on knowledge of the source of the hazard prior to implementation. For example, concern for insufficient control measures may be assessed at a higher level for a foodborne outbreak with no known source, because deployment of a food recall, an effective control measure, is dependent on knowledge of the source of the outbreak.

### Overall threat assessment

The overall threat assessment provides a single assessment that communicates the threat level, allowing decision-makers to better understand the degree to which public health action is needed for a given threat. All three attributes are taken into account when determining the overall assessment, although they may not receive equal weight. As outlined in **Table 3**, a pathogen associated with severe or fatal outcomes may receive a low overall assessment if it is unlikely to affect Canada and can be effectively managed should it occur. Recognizing the subjectivity of this approach, the final overall assessment level is made by group consensus during SCCTA meetings.

**Table 3:** An example using Public Health Agency of Canada’s threat assessment method

Situation summary			
Public Health Agency of Canada was notified that the Ministry of Health from Country X has reported an outbreak of hemorrhagic fever, including several deaths. The majority of cases and deaths are among health care workers from two health facilities. Additional contacts have been identified and are under follow-up. Transmission of the virus requires close contact with body fluids of an infected individual. The Government of Country X is coordinating the response, including case finding, contact tracing, implementation of infection prevention and control at health facilities, risk communication, and assessment of possible vaccines and therapeutics for clinical trials. Country X has also implemented border control measures to prevent potential cases from travelling outside the country. No approved therapeutics or vaccines exist. Public Health Agency of Canada programs have consulted travel data and find that the monthly number of travellers between Canada and Country X in the following three months is estimated to be low.			
Threat attribute	High	Moderate	Low
Concern for human health	☒	☐	☐
Potential to affect Canada	☐	☒	☒
Concern for insufficient control measures	☒	☐	☒
Overall threat assessment	☐	☐	☒
Rationale for the threat assessment level			
The overall threat level is assessed as low for Canada at the time of assessment. The concern for human health is assessed as ‘high’ because Disease X is a severe, often fatal illness in humans. The case fatality ratio ranges from 24%–88% depending on the virus strain and case management. The potential to affect Canada is assessed as ‘low’ because there are no cases in Canada and Country X has implemented appropriate infection prevention and control measures, including border measures, to prevent the spread of the virus outside of the affected region. In addition, travel volumes between Canada and Country X are relatively low. The concern for insufficient control measures is assessed as ‘low’. There are no vaccines or treatments approved for this virus however, Canada has sufficient capacity for detection of cases and measures to limit spread if a travel-associated case is detected.			

### Rationale for the overall threat assessment

A narrative rationale for the overall threat assessment level provides the context within which each threat is assessed and supports the assigned threat level based on the information used to make the assessment. The rationale describes the level of each individual threat attribute, and the way it contributes to the overall assessment of the threat. This includes any nuance in the reasoning behind the level selected for the individual threat attributes and overall threat assessment, key information gaps, and associated uncertainty related to the assessment.

## Discussion

The coordinated threat assessment process at PHAC was created in response to recommendations from the Auditor General of Canada’s 2021 report on pandemic preparedness, surveillance, and border measures following the COVID-19 pandemic ((7)). The objective of PHAC’s coordinated threat assessment process is to produce a quick, timely snapshot of threats for PHAC senior leadership using standardized methodology. This process provides a systematic scientific approach to assess varied threats from across PHAC programs by consolidating emerging data and SME opinion on a weekly basis. The process established at PHAC optimizes the ability to anticipate, detect, understand, and respond to potential public health threats through communication of timely assessments and corresponding actions for public health partners within PHAC and across Canada.

While different public health agencies vary in their processes and hazard priorities, many agencies employ a standard tool to characterize and assess public health threats ((2–5)). Public Health Agency of Canada’s standardized threat assessment tool reflects three key elements to consider in terms of public health significance, ensuring a consistent methodology is applied to a wide variety of threats with transparent outcomes.

### Limitations

A critical principle of the threat assessment framework is that the application of the threat attributes is qualitative and flexible and can be applied to any public health threat of concern to Canada. Flexibility in the application of the framework is key, given the breadth of data and intelligence that may be used to inform the assessment. For example, an event associated with a pathogen will use different indicators to assign a level to individual threat attributes compared to an event associated with a natural disaster. The former may characterize concern for human health using the case fatality ratio, whereas the latter may rely on burden of harm to mental health. The rationale for the threat assessment provides context for the selected level and ensures transparency in the assessment regardless of the indicators used to arrive at the assessment level.

The qualitative nature of the threat assessment definitions can make it challenging to assign a level of ‘high’, ‘moderate’, or ‘low’ for each section without quantitative guidelines. However, this subjectivity allows for expert opinion influencing the assessment of threats and flexibility in weighting threat attributes based on the context of the event. The approach also allows for discordant opinions, which are discussed during SCCTA meetings and resolved by group consensus.

Some public health agencies take an all-hazards approach ((2)), while others are focused on infectious diseases ((3,4)). At the time of writing, the majority of threats assessed by PHAC have been related to infectious diseases, however, a small number of non-infectious disease threats have been submitted to the threat report (e.g., environmental hazards, mis/disinformation). While such signals are often the responsibility of other government departments, there is ongoing work to expand the scope of the CTA process to include a broader spectrum of health hazards ((15,16)). Recognizing the increasingly interconnected and complex nature of natural and human-induced hazards and their potential impact on health ((16)), an approach that includes infectious and non-infectious hazards increases efficiency by recognizing and integrating common threat assessment elements across hazard types. Accordingly, the threat assessment framework described in this paper was designed with an inclusive approach in mind and is expected to remain applicable in assessing threats associated with diverse hazard types.

## Conclusion

In summary, public health intelligence, including threat assessment, is a key component of public health infrastructure. Early assessment of threats can be challenging with limited and emerging information, however, a standardized methodology ensures all public health threats are characterized using a consistent approach. The implementation of CTA across PHAC has improved the process and transparency of risk assessment activities and fosters relationships by sharing results with public health partners.
